# Comprehensive Analysis of Two H13-Type Starting Materials Used for Laser Cladding and Aerosol Particles Formed in This Process

**DOI:** 10.3390/ma15207367

**Published:** 2022-10-20

**Authors:** László Péter, János Osán, Szilvia Kugler, Veronika Groma, Simone Pollastri, Attila Nagy

**Affiliations:** 1 Wigner Research Centre for Physics, P.O. Box 49, H-1525 Budapest, Hungary; 2 Centre for Energy Research, P.O. Box 49, H-1525 Budapest, Hungary; 3 Elettra Sincrotrone Trieste, Basovizza, I-34149 Trieste, Italy

**Keywords:** additive manufacturing, laser cladding, H13 alloys, aerosol composition, X-ray analysis

## Abstract

Laser cladding with H13 steel powders was performed and the related material transformations were studied for the particles emitted during this process. Fractions of various sizes of the aerosol particles formed during the laser cladding were collected on a cascade impactor, while the electromobility and the aerodynamic size of the particles were measured using a scanning mobility particle spectrometer and an aerodynamic particle sizer, respectively. The aerosol particles deposited onto the impactor plates were analyzed using scanning electron microscopy–energy-dispersive X-ray spectroscopy, as well as total-reflection X-ray fluorescence and X-ray absorption near-edge structure spectroscopy. Both the concentration and mean oxidation state of the major components were correlated with the aerosol particle size. The ultrafine aerosol particles (with a diameter less than about 100 nm) were predominantly oxidized and formed as the result of an evaporation–oxidation–condensation process sequence. The larger particles (>200 nm in geometric diameter) were primarily the residues of the original metal powder and exhibited a composition change as compared to the as-received metal powder. Correlations between the changes in the concentration ratio of the components were detected and explained.

## 1. Introduction

Understanding the processes involved in intense laser light–metal interactions is of paramount importance for the application of lasers in industrial technologies such as laser welding and 3D metal printing. While studies of these processes provide essential information for modelling and technology development [1,2], other aspects give insight into occupational health-related issues [3,4,5], similarly to the conventional welding processes [6,7].

The direct energy deposition (DED) technique is a rapidly developing additive manufacturing technology [8,9]. It is widely used to either repair or produce parts to create a cladding on an existing surface to enhance its corrosion resistance, hardness, or wearing properties. In a laser-based DED machine, the energy source melts the substrate where the powder particles are deposited, creating a new layer. The cladding on the surface of the object is then created layer-by-layer. At the point of interaction with the laser, the metal melts and the high-temperature melt pool begins to evaporate. Simultaneously, a large number of nanoscale particles are formed from the hot metal vapor. These primary particles then aggregate or agglomerate to form larger particles that can penetrate deep into the lungs. The nanoparticle emission of laser-DED machines has been recognized, and some studies have already addressed the chemical and physical characterization of the emitted particles [10] and investigated occupational health-related issues [5,11]. The toxic properties of metal nanoparticles are now well known [5,12,13,14,15,16,17,18] and their effects need to be taken into account in working environment design and laser-DED machine construction. Although exposure protection and regulatory measures in related industries (welding, etc.) are applicable to DED technology, it is crucial to characterize the properties of aerosol particles formed in this process. In addition, it has already been shown that the composition of the emitted particles changes as compared to the as-received metal powder [19]. This phenomenon has also been observed in similar processes with laser ablation, where short-pulse lasers (nanosecond to femtosecond pulses) are used to irradiate different materials to produce nano- to microscale particles [20,21,22,23]. All of this information will help to develop appropriate occupational health measures for such technologies.

H13 alloy is a hot-work tool steel that belongs to the group of steel alloys with typical applications in the fields of hot die work, die casting, and extrusion dies. The chromium content helps this alloy to resist softening at higher temperatures. Besides iron and chromium, it also contains molybdenum and vanadium as strengthening agents. This material type has high hardness and corrosion resistance; furthermore, it can be well polished in the bulk form [24,25]. The machinability and ductility properties of H13 are favorable; hence, it can be formed via conventional means. These properties, together with its melting point (1427 °C) and near-infrared absorption properties, make it an ideal material for additive manufacturing.

The H13-type starting powders for laser cladding are produced using a gas atomization process. This is a widely used technique for powder production and may introduce irregularities and satellite particles to the mainly spherical powder, depending on the production parameters [26,27]. The irregularities affect the apparent density and flowability of the powder and the delivery of the powder particles to the point of laser interaction [28]. Satellite particles can remain in the ambient air, causing health problems and affecting the process itself [29].

In this article, the properties of two kinds of H13 steel powders and their aerosol products formed in the laser cladding process are compared. Size-fractionated aerosol samples were collected using a cascade impactor on silicon and adhesive carbon substrates, in parallel to which the electromobility and aerodynamic size of the particles were measured using a scanning mobility particle spectrometer (SMPS) and an aerodynamic particle sizer (APS), respectively. The morphology and chemical composition of both the powder particles and the collected aerosol samples were studied via scanning electron microscopy–energy-dispersive spectroscopy (SEM-EDS). The size dependence of the elemental concentrations and the oxidation degree of metallic elements in the collected aerosol particles were investigated using a total-reflection X-ray fluorescence (TXRF) analysis and X-ray absorption near-edge structure (XANES) spectroscopy, respectively. This combination of complementary microanalytical techniques was selected in order to characterize aerosols at the individual particle level (SEM-EDS), as well as for a collective analysis of size-fractionated aerosol particles from samples of microgram amounts (TXRF and XANES).

The aim of the present work was to clarify the processes leading to the formation of aerosol particles during the laser cladding process, so as to characterize their size distribution and identify the composition and oxidation state changes taking place in the aerosol particles compared to the as-received metal powder.

## 2. Materials and Methods

### 2.1. Powders Used in the Laser Cladding Process

**Figure A1:**
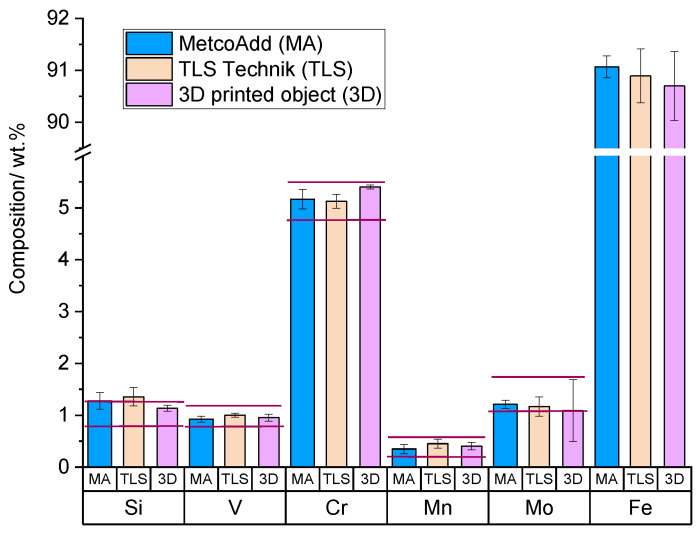
Composition of the materials studied. The left and middle columns for each element show the starting materials, while the right column refers to the welded objects. Horizontal lines for the minor components show the standard composition requirement for H13 steel products (see ASTM A681; also see Table 1 of the main text). Error bars show the standard deviations of 4 measurements made for each sample type.

For the laser cladding processes, H13 steel feedstock powders produced by Oerlikon (MetcoAdd H13-B) and TLS Technik were used. Both tool steel powders have a size range of 45 to 90 µm, mostly have a spherical or ellipsoidal form, and were produced by gas atomization manufacturing process. Table 1 shows the nominal composition of the hot-work tool steel according to different standard documents used worldwide. It is to be noted that the specifications of the manufacturers of the powders used in the present study indicate much wider composition ranges (up to a few tens of % in some cases), but their actual composition is essentially conformal to either of the standards (see Appendix A, Figure A1).

### 2.2. Additive Manufacturing

**Scheme 1:**
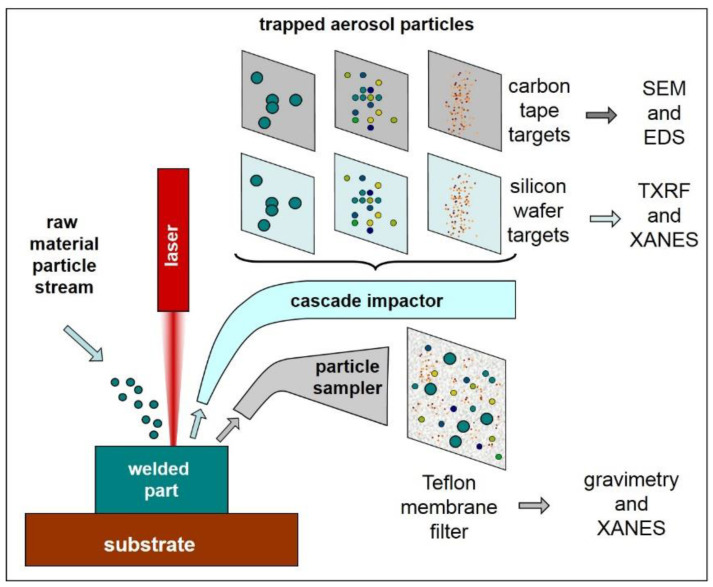
Scheme of the apparatus used for laser welding, the sets of samples collected, and the analysis methods by which the specific samples were studied.

A laser-based open bench-top-directed energy deposition additive manufacturing machine was used for the study. The LRS EVO-Diodeline 450 system (OR Laser Technology Inc., Dieburg, Germany) was described in a previous study in detail [19], so only its main features are summarized here. The laser source is a single-mode randomly polarized diode-pumped YLM ytterbium fiber laser (IPG Photonics, Oxford, MI, USA) with 1070-nm-wavelength infrared radiation and a 450 W maximum mean output power. The laser was used in continuous wave mode during the experiments. The output power is adjustable between 10 and 100%, and the beam diameter at the surface of the workpiece is adjustable between 0.48 and 3 mm. The laser’s measured output power was 361 W (80% power) and the spot diameter was 1.04 mm in the experiments. A 200 mm focusing optics piece is integrated into the powder nozzle, which delivers the powder and the shielding Ar gas to the working point. The system is fed with the metal powder by a GTV PF2/1 LC powder feeder (GTV Verschleißschutz GmbH, Luckenbach, Germany) with argon at a 3.8 L/min carrier gas flow rate and 5.3 g/min powder mass flow rate. During a typical laser cladding cycle, the additive machine was used to build demo rectangles measuring 15 × 15 × 5 mm^3^ (width × length × height) on a 304 L stainless steel substrate. The scheme of the apparatus and the set of samples produced or collected during the laser welding process, together with the analysis results, are depicted in Scheme 1.

### 2.3. Aerosol Collection and Monitoring Devices

Size-fractionated aerosol samples were collected on either bare Si or adhesive carbon substrates using an in-house developed extension of the May-type cascade impactor [33]. The impactor separates particles into nine size fractions according to their aerodynamic equivalent diameter. The cut-off sizes of the impactor stages for the gas flow rate used are given in Table 2. The cut-off size refers to the aerodynamic size at which 50% of the particles are captured on a given impactor stage. Particles with diameters >~20 μm having the same density as the original feed powder are sedimented from the air within seconds, which prevents them from entering the air stream channel leading toward the impactor plates. Therefore, the impactor plate with the cut-off sizes given in Table 2 can catch essentially all particles that are capable of staying in the air as aerosol particles for longer than 10 s.

The sampled particulate matter was deposited as a thin stripe (0.1 mm to 1 mm wide, 50 mm long) along the axis of symmetry parallel to the longer edge of each rectangular impaction plate. Due to the high aerosol concentrations measured in the laboratory during the laser cladding process, the sampling time was kept short. The collection of aerosol particles was sequential for both feedstock powders. Sampling on Si substrates for 10 min was followed by a separate sampling phase on adhesive carbon substrates for 40 min. Simultaneously with the sample collection on the cascade impactors, the total suspended particles were collected on Teflon membrane filters (47 mm diameter and 1 µm pore size) using a 60 L/min flow rate. The building of a new demo rectangle was started at the beginning of each sampling phase. Prior to each sampling phase, the concentration in the room reached the background level due to the extractor device, which was three orders of magnitude lower than during sampling according to the SMPS data.

The numerical concentration and number size distribution of the aerosols were monitored via SMPS and APS during each cascade impactor sampling phase (with the SMPS+C Model 5416 device from Grimm Aerosoltechnik Ltd., Ainring, Germany and the APS Model 3321 device from TSI Incorporated, St. Paul, MN, USA, respectively). While the APS measures the aerodynamic diameter, like the cascade impactor, the SMPS determines the electrical mobility diameter of the particles. The two types of equivalent diameters can be different from each other based on the shape and density of the particles [34]. The two instruments together cover wide size ranges with only a small region of overlap, with 10 to 1094 nm and 0.5 to 20 µm for SMPS and APS, respectively.

During the laboratory experiments, inlets of the sampling and monitoring equipment were placed 80 cm away from the source of the laser-cladding-generated aerosols, which is the typical distance of the operator’s head from this point for the machine type used.

### 2.4. Scanning Electron Microscopy and Energy-Dispersive Analysis

A TESCAN MIRA3 type scanning electron microscope (SEM; manufactured by TESCAN/Brno, Czeck Republic) equipped with a field-emission cathode was used for imaging both the original particles and the products collected on the impactor plates. The composition of the particles was analyzed with an EDAX Element-type energy-dispersive X-ray spectrometer (EDS) attached to the same SEM device. For the morphology analysis, secondary electron images were recorded. The composition analysis was performed with a 20 kV acceleration voltage. At this voltage, all X-ray lines necessary for the analysis could be obtained with a sufficient yield. For the original particles measuring several tens of micrometers in diameter, the penetration depth of the electron beam was much smaller than the particle size, meaning they could be considered as bulk materials for the purpose of the SEM-EDS composition analysis. Therefore, their composition will be given below as provided by the software used for the SEM-EDS analysis. For the mean composition of the aerosol product, the above-mentioned practice was followed, although it is known that the total thickness of the deposited aerosol particles is often less than the penetration depth of the electron beam. For the analysis of the individual particles, however, a normalization process was applied in which the characteristic net X-ray intensity (I) of the minor components (Mc) was divided by that of the major component (Fe), and this ratio was divided by the same quantity derived from the spectrum of the starting material (SM):

x*_Mc_ = (I_Mc_/I_Fe_)/(I_Mc,SM_/I_Fe,SM_). (1)

Here, x*_Mc_ is a normalized intensity that is analogous to the ratio of the amount of the minor component to the selected major component (Fe). The reason why the analogy with the component ratio is incomplete lies in the fact that the electron collision cascade and the concomitant X-ray emission pattern are highly dissimilar in bulk materials and small particles.

### 2.5. Total-Reflection X-ray Fluorescence Analysis

The elemental composition of aerosol samples collected on Si substrates was determined for each impactor stage via the total-reflection X-ray fluorescence (TXRF) analysis. A compact laboratory TXRF system [35] was applied for the present study. The sensitivity of TXRF for the elements to be detected as the components of the H13 type materials has been well demonstrated in aerosol studies [36]. Our TXRF system consisted of a 50 W microfocus Mo-anode X-ray tube (Petrick, Bad Blankenburg, Germany), a Mo/Si multilayer monochromator (AXO, Dresden, Germany), and a 7 mm^2^ silicon drift detector (KETEK, Munich, Germany). The system was operated at 50 kV and 1 mA, and Mo-Kα X-rays were used for excitation. The measurements were performed in the air in a geometry with an incoming beam direction perpendicular to the aerosol deposit stripe, with counting times of 1600 s (stage 9) and 3000 s (stages 8–3). The X-ray spectra were evaluated using the AXIL software [37]. The quantification of the elemental content was performed using external calibration based on reference materials (Merck IV multielemental solution, Cr pads with a stripe-like deposition geometry [38]); details can be found in our previous study [39]. The detection limit of 100 pg/m^3^ at each impactor stage was reached using the system for transition metals in ambient aerosol particles [39], assuring the reliable determination of elemental size distributions for laser-cladding-originated aerosols [19].

### 2.6. X-ray Absorption Near-Edge Structure Spectroscopy

X-ray absorption near-edge structure (XANES) measurements were performed at the XAFS [40] and XRF [41,42] beamlines of the Elettra synchrotron radiation facility (Trieste, Italy). The XANES spectra were collected at the K absorption edges of Cr, Mn, and Fe using a Si(111) monochromator at both beamlines. At the XRF beamline, the spectra from size-fractionated aerosol particles deposited on Si wafers were measured in the TXRF geometry using an XFlash 5030 SDD (Bruker, Berlin, Germany). At the XAFS beamline, the geometry for fluorescence-mode measurements on filter samples was a standard 45°/45°, using an AXAS-M SDD (KETEK GmbH, Munich, Germany) detector. Reference XANES spectra were collected in transmission mode (sample at 90° with respect to the beam). Pure Cr, Mn, and Fe metallic foils and pressed pellets of pure chemicals (Cr_2_O_3_ and Fe_2_O_3_) were used as standards for the relevant oxidation state of a specific element.

All spectra were collected at room temperature both in the air (XAFS beamline) and in high-vacuum (XRF beamline) conditions, using a variable energy step as a function of the energy: a large step (5 eV) in the first 200 eV of the spectrum, a smaller step (0.2 eV) in the near-edge region, and a k-constant step of 0.03 Å^−1^ further above the absorption edge. The time per step was 5 s for fluorescence and 2 s for transmission-mode measurements.

For each sample, multiple spectra were collected and merged in order to increase the signal-to-noise ratio. The oxidation state of the metals in the aerosol samples was determined using least-squares linear combination fitting (LCF) based on reference spectra collected for model compounds of known oxidation states. The background removal, normalization of XANES spectra, as well as LCF were performed using the Athena software package [43].

## 3. Results and Discussion

### 3.1. Comparison of the Particle Size, Particle Morphology, and Composition of the As-Received Powder Samples

**Figure A2:**
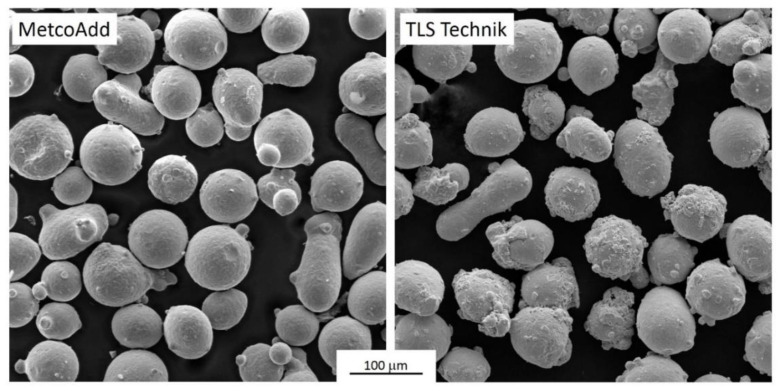
Representative scanning electron micrographs of the as-received H13 particle assemblies attached to adhesive carbon tapes.

**Figure A3:**
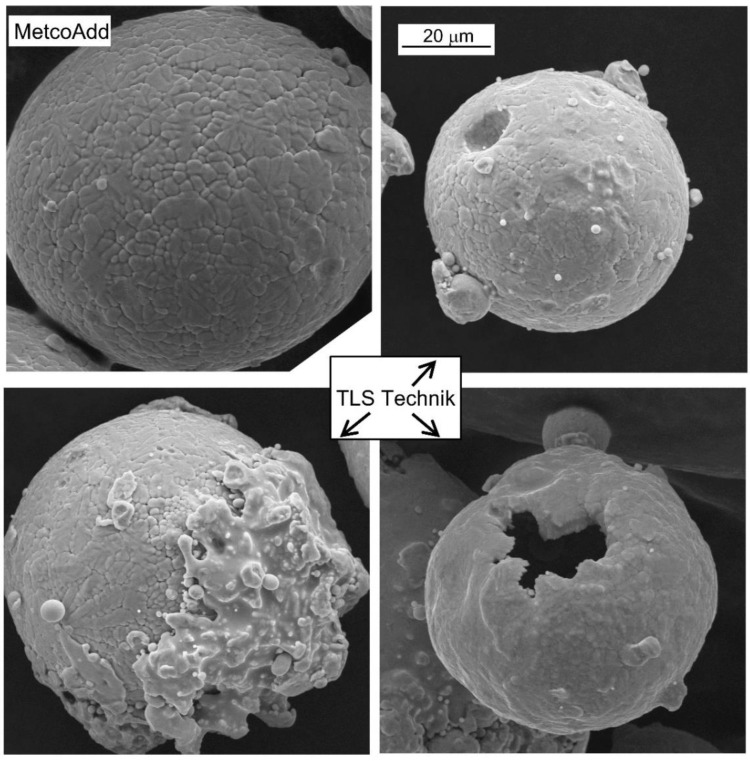
Types of particles found in the powder populations used in the present study.

**Figure A4:**
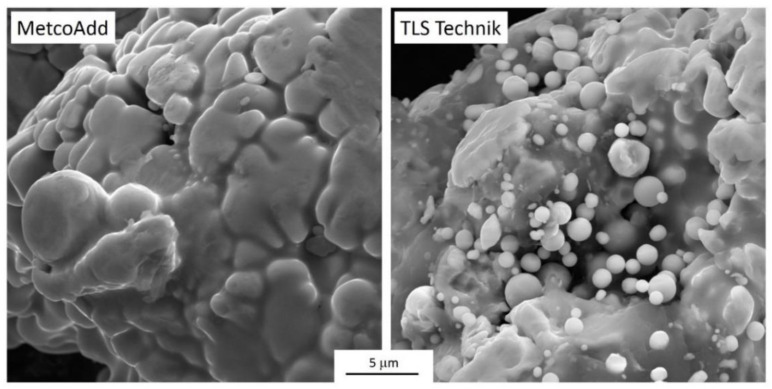
High-magnification SEM images of the surfaces of the truncated parts of the particles for both powder types.

The size and morphology of the original particles were studied in detail (see Appendix B, Figure A2, Figure A3 and Figure A4). Both batches proved to be fully conformal to the standards of the H13 allow to be used for laser-welding-based additive manufacturing processes. The morphological study revealed subtle differences that had no relevance for the forthcoming analysis, considering that the large majority of the particles were melted during the laser welding process. The only difference with a consequence on the aerosol formation was that the surfaces of the TLS Technik particles were often decorated with loosely attached spherical objects measuring <3 µm in diameter (see the discussion later in Section 3.2).

The as-received metal powders were of nominally identical composition and conformal to the H13 steel type. Although the Si contents of both products were somewhat larger than the limit, the deviation was still within the precision range of the SEM-EDS analysis. The nominal carbon content of H13 steel is 0.32–0.45 wt.%. However, carbon and oxygen were omitted from the composition analysis because they are common impurities of the vacuum system, and their bulk composition level cannot be measured accurately with SEM-EDS due to impurity adsorption. Apart from the metals shown, Ni and Cu are also allowed to be present in H13 steels in accordance with the ASTM standard (up to 0.3 and 0.25 wt.%, respectively). However, their concentration in the raw material was so small that their EDS lines were not observed. The compositions of the as-received powders and the final printed object samples are shown in Appendix A (Figure A1).

### 3.2. Shape, Size Distribution, and Composition of the Particles in the Fume Collected by the Cascade Impactor during the Laser Cladding Process

**Figure 1:**
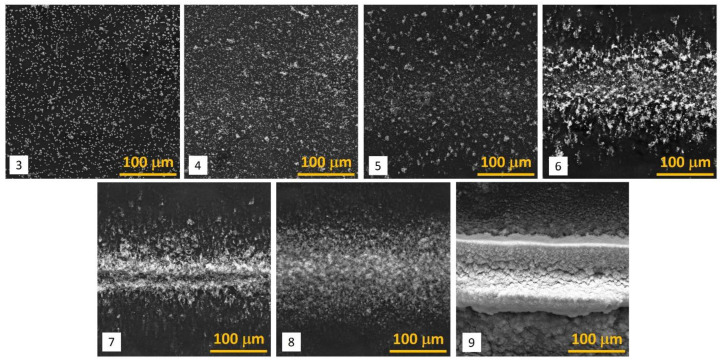
Low-magnification SEM images of the surface of the impactor plates after the particle collection. Numbers at the bottom left corner of each image indicate the impactor stage identifier as shown in Table 2. Starting material: TLS Technik H13 powder; laser operation power: 80–85% (the image set is representative for both starting materials and all laser powers tested).

**Figure 2:**
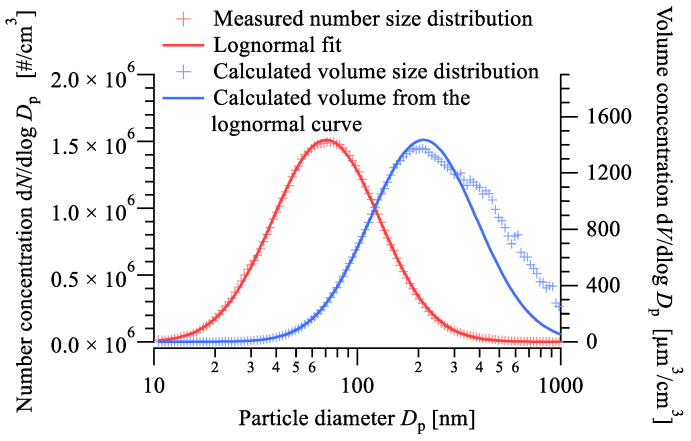
Measured size distribution of the released particles by the SMPS just after laser cladding with the TLS H13 powder. The background concentration in the room was three orders of magnitude lower than the concentration of the new particles. The measured size distribution can be modeled by a lognormal distribution. The blue markers and the blue line show the calculated volume size distributions. The line is calculated from the lognormal fit, while the markers are calculated from the measured data.

The low-magnification SEM images of the carbon substrates with the impacted particles can be seen in Figure 1. The analysis of the first two stages of the impactor is omitted because of the low amount of deposited material. The negligible amount of aerosol particles shown on impactor plates #1 and #2 is related to the very fast sedimentation of the relatively large particles that fall down prior to entering the air stream toward the analyzer, meaning they cannot reach the impactor plates. The deposit collected on impactor stage #3 is composed of relatively large particles that fill the impactor plate surface rather randomly. As one proceeds to impactor stages with a higher number, the individual particles become less and less discernible, and a foamy fume-like deposit is obtained that is situated at the central line of the impactor plate. From the visual observations, the largest amount of deposit was obtained at the impactor stage that collected the smallest particles. This is in line with the results of the SMPS measurements (see Figure 2).

**Figure 3:**
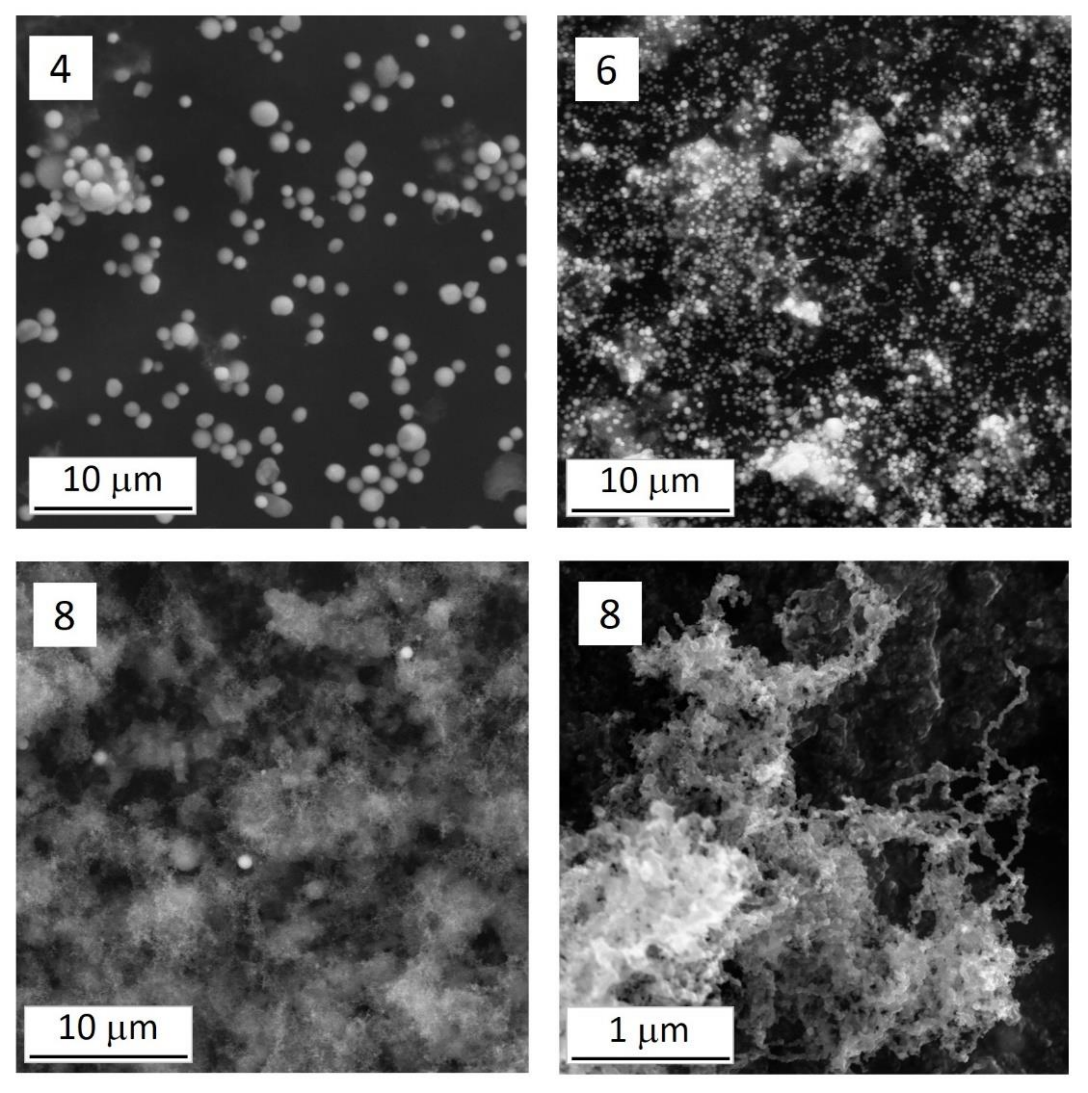
High-magnification SEM images of the surfaces of a few impactor plates. Numbers at the top left corners of each image indicate the impactor stage number as defined in Table 2.

**Figure A5:**
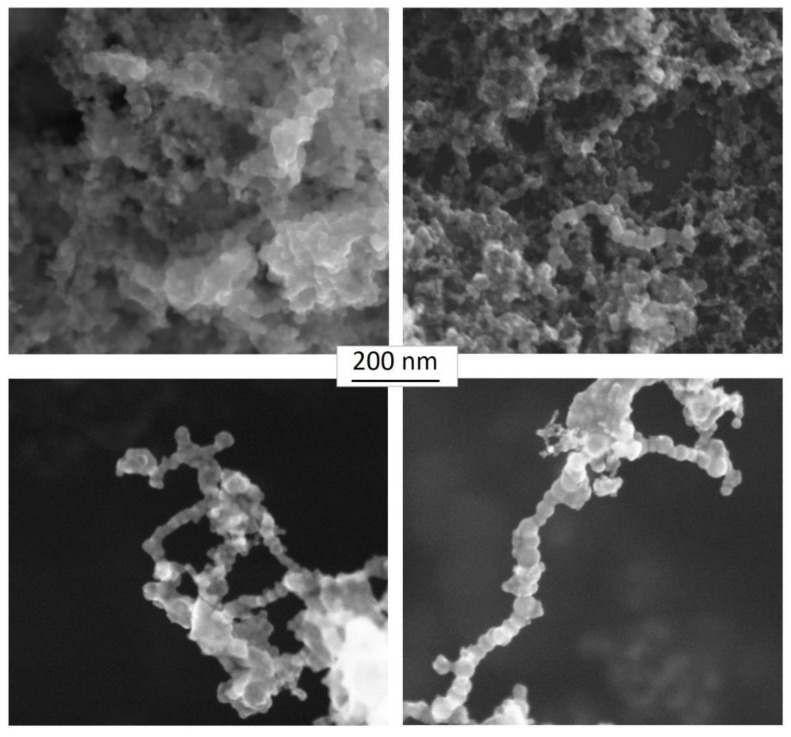
High-magnification SEM images of the deposits formed on the impactor plates. Top images: Thin deposit layers where the primary particles can be identified. Bottom images: Filaments composed of the primary particles and reaching far from the impactor plate.

Figure 3 presents characteristic areas of a few impactor plates at higher magnifications. It is noteworthy that the particles observed at lower impactor stages are smaller than the particle sizes associated with these stages (for example, on impactor stage #4; see Figure 3). Therefore, it was concluded that the particles travelling toward the impactor are likely to form aggregates of a few particles that fall apart upon the collision with the impactor plate. At higher impactor stages, one can see that the spherical particles serve as accumulation or nucleation centers for ultrafine particles. Where the deposited particles form a thin layer at the rim, the morphology of the individual ultrafine particles can be scrutinized as well. Compact globular units with a diameter as small as 20 nm were found in many cases, and these particles are often organized into filaments (Figure 3, bottom right image; for further high-resolution SEM images, see Appendix C, Figure A5). At positions where the deposit is thick, the filamentary structure cannot be identified, but the images show a fume-like cloudy pattern due to the random superimposition of the particle layers.

Taking into account the density of the metallic powder investigated here (7.8 g/cm^3^), the geometric size of the particles observed by SEM on the impactor plates is in good agreement with the theoretical aerodynamic diameter range of the respective stages.

The ratio of the particles with relatively large and small diameters (>≈70 nm and <≈25 nm, stand-alone and filament-forming ones, respectively) depends on the laser power applied. The lower the laser operation power, the larger is the ratio of the relatively large individual particles in the deposit. This is indirect evidence that the ultrafine particles are formed in an evaporation–precipitation process, which is less effective with reduced laser operation power.

**Figure 4:**
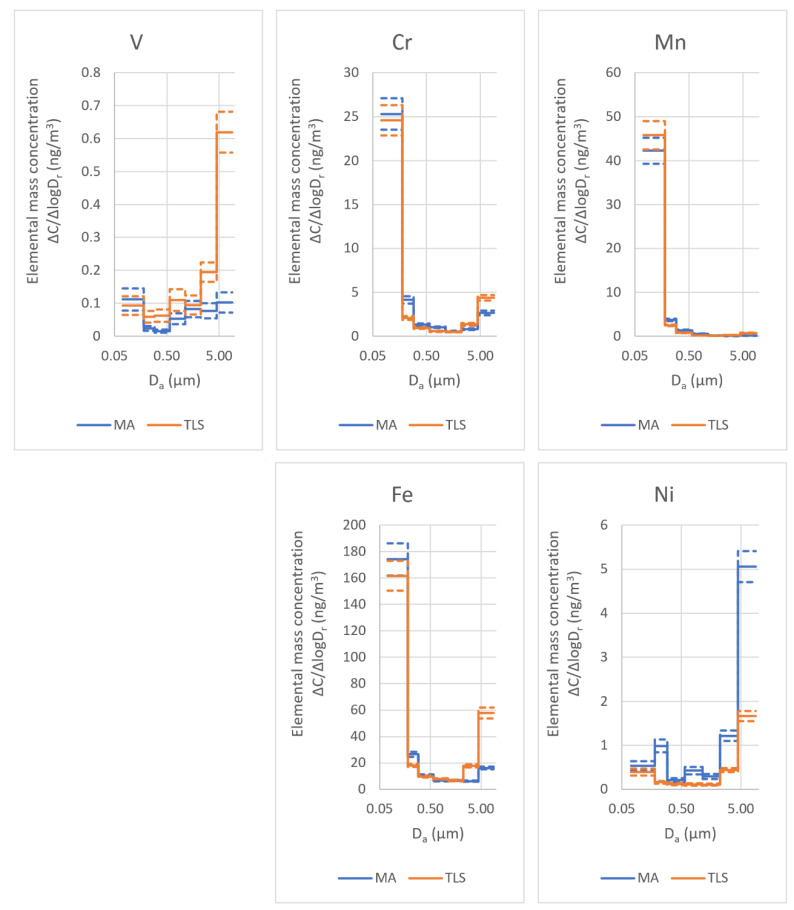
Size distribution of particulate V, Cr, Mn, Fe, and Ni concentrations for samples collected during laser cladding operation using two different H13 feedstock powders (MetcoAdd—MA; TLS Technik—TLS). Uncertainties (±σ) are indicated using dashed lines.

In addition to the number size distribution obtained by simultaneous SMPS measurements, the size distributions of elemental concentrations could be calculated from the air concentrations for each size fraction determined via TXRF. In line with the expected lognormal distribution, the size distributions are presented as ΔC/ΔlogD_r_ in µg/m^3^ for major and minor metallic components present in the particulate matter formed from H13 materials during laser cladding (Figure 4). Since the argument of the log function must be dimensionless, D_r_ denotes the relative particle diameter (D_r_ = D_a_/D_0_, where D_a_ is the aerodynamic equivalent diameter and D_0_ = 1 µm).

The aerosol concentrations of the main element Fe show a bimodal size distribution in both sample sets (Figure 4, panel Fe), with a major peak in the ultrafine fraction and a less pronounced peak in the coarse fraction. The most pronounced ultrafine mode is also reflected in the size distribution of the total number (see Figure 2) and the low-magnification SEM images of the impacted stripes (see Figure 1), as most of the particles were generated in the ultrafine size range. The ultrafine peak is of a similar magnitude for the two kinds of H13 feedstock powders, while the coarse peak is three times higher for H13 TLS Technik than for H13 MetcoAdd (Figure 4, panel Fe). Coarse airborne particles were most probably detached from the surfaces of the original grains of the feedstock powders, which might also interact with the laser beam. The difference in the Fe concentrations in the coarse fraction is in line with the SEM observations of the surfaces of the grains of the as-received metal powders, showing that in contrary to the MetcoAdd powder, the TLS Technik powder contains almost perfectly spherical satellite particles measuring <3 μm in diameter (see Appendix B, Figure A4).

Among the major constituents of the original H13 tool steel (also shown in Appendix A, Figure A1), vanadium is below the detection limit at impactor stages 6 through 9 as measured by SEM-EDS. The TXRF results show only a minor content of vanadium in the fine fraction, indicating a great depletion by a factor of ≈20, while the coarse particles contain almost the same amount of vanadium as the original powders (see Figure 4, panel V). As we demonstrated in our previous work [19], the components selectively evaporate from the original particles and the governing factor of the component loss is the vapor pressure of the components. The final composition of the aerosol is obtained as a result of an evaporation–oxidation–condensation process sequence, leading to both the depletion of volatile components in the residues of the large particles and the accumulation of easily oxidizable components in the fine particle fraction. Hence, it can be understood that vanadium (i.e., the component with the largest vapor pressure) is nearly completely leached out from the particles (for vapor pressure and oxidation heat data, see Appendix D). However, the surprising fact is that it was not redeposited in the aggregate. The explanation could be that due to its relatively large vapor pressure, it can diffuse far away from the aggregate formation zone during the welding process, meaning it cannot be collected with the air stream. It is also possible that it also takes part in the formation of aggregates, but the vanadium-containing particles are so small that they cannot be trapped in the impactor system. In either of the above-mentioned cases, ultrafine vanadium-containing particles (probably oxides) may impose a massive health risk if inhaled.

Since all components of the original metal powders are somewhat volatile under the circumstances of the laser cladding, there is no fixed point to which the rest can be referred to. Nevertheless, since Fe is the major component, we chose it as a reference component, even though it can also be evaporated and then partly aggregated. By referring to Fe, it can be seen that the concentrations of Si, Cr, and Mo as obtained by SEM-EDS were somewhat larger in the overall deposit on each impactor plate than the corresponding amounts in the as-received metal powders. Nevertheless, the data obtained were very scattered and did not show a pronounced trend across the impactor plate series. The element ratios were as follows: Fe/Si: 20–60 (original: ≈65), Fe/Cr: 8.5–24 (≈18.2), Fe/Mo: 20–53 (≈82). This is in accordance with the order of the volatility of these components. In line with the SEM-EDS results, Cr shows two-fold enrichment at stage 9 (70–180 nm fraction) and slight depletion at stages 3 and 4 (2.5–8.9 µm fraction) (see Figure 4, panel Cr).

**Figure 5:**
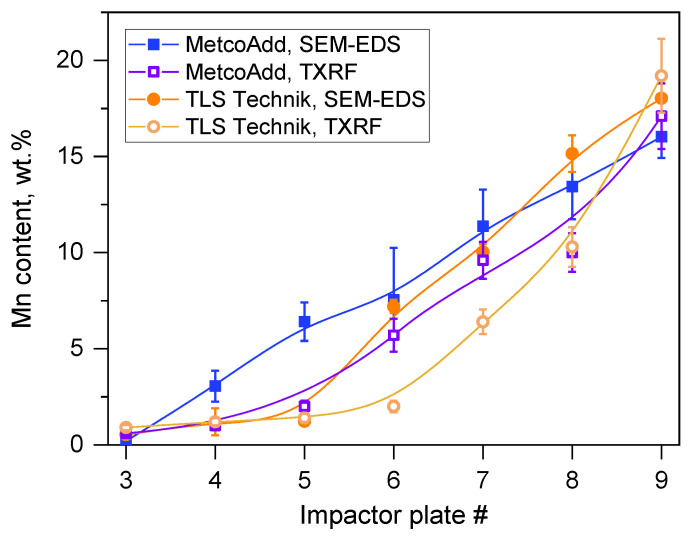
Manganese weight percentages as measured using SEM-EDS and TXRF for large surface areas of the impacted particle deposits. Sampling on the Si substrates for the TXRF analysis was followed by separate sampling on adhesive carbon substrates for the SEM-EDS analysis. The error bars indicate the standard deviation of 4 measurements.

In contrast to Si, Cr, and Mo, well-detectable Mn accumulation was found via both SEM-EDS and TXRF at all aerosol deposits collected at the impactor surfaces. The Mn fractions, as referred to for the other original components, varied systematically across the impactor series (see Figure 5), and the values measured were all significantly higher than that in the as-received metal powders (≈0.5%). The size distributions of the air concentrations (see Figure 4, panel Mn) show that Mn is so enriched in the smallest particles collected by the cascade impactor that the Mn concentration becomes even larger than that of Cr.

The significant Mn accumulation cannot be explained merely by the volatility order of the components. Rather, the strong inclination of Mn to become oxidized may significantly contribute to the Mn accumulation, considering that the vapor pressures of the oxides are generally much smaller than those of the parent metals. The latter factor also explains the fact that a large Mn excess could be seen for the small aerosol particle sizes (i.e., at high impactor numbers) [19]. As is shown in Appendix A of Figure A1, no Mn loss can be seen when the composition of the welded deposit is scrutinized. This is well understood, since the weight of the welded deposit is several orders of magnitude larger than that of the aerosol produced.

Foreign elements were occasionally detected via SEM-EDS as deposit components on the impactor plate. The cumulated concentration of Ni, Cu, and Zn is always less than 1.5 wt.%, and their appearance is probably related to either the composition or impurity profile of the welding platform or the additive powder previously used in the laser welding device. It is typical that these components are either missing entirely on the impactor plates used in a particular experiment or present at each of the impactor stages. The TXRF results for the Ni concentrations (see Figure 4) show a maximum in the coarse fraction (stage 3). Ni-rich individual spherical particles were also detected via SEM-EDS at stage 3, which might be reminders of the previously used Ni-alloy feedstock powders.

The total air concentrations of major elements in aerosol particles considering impactor stages 3–9 were found to be 89–99 µg/m^3^ for Fe, 18–20 µg/m^3^ for Mn, and 12–13 µg/m^3^ for Cr for both H13 starting materials. These results are in accordance with those reported for conventional welding processes [6], although corrosion-resistant steels and construction steels were studied covering a wide range of Cr contents as compared to H13 tool steel. The previous results also indicated a strong enrichment of Mn in the fumes compared to the welded steels.

**Figure 6:**
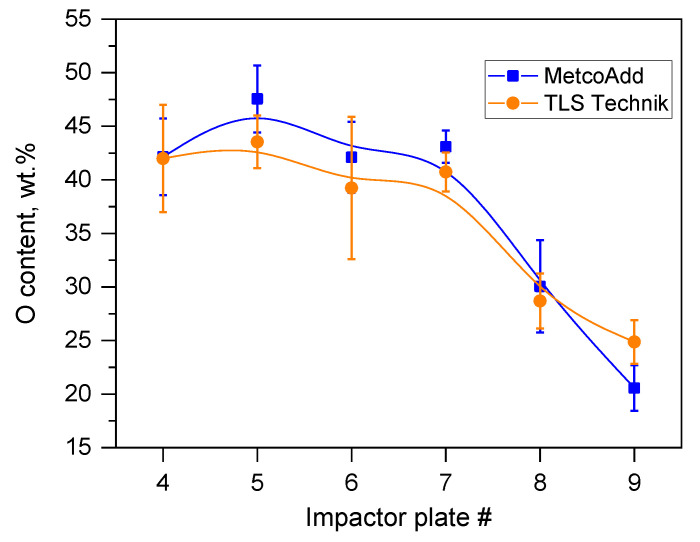
Oxygen weight percentages as measured via SEM-EDS on large surface areas of the impacted particle deposits. The carbon contribution originating from the impactor was omitted from the EDS results, and the rest of the components were normalized. Error bars indicate the standard deviations of 4 measurements.

**Figure A6:**
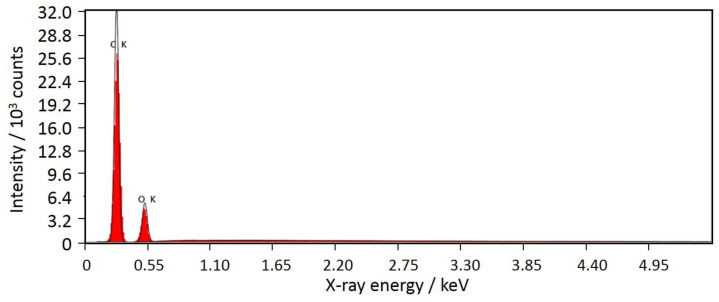
SEM-EDS spectrum of the carbon substrate used for the impactor plates.

The particles trapped on the impactor surface are oxidized to various extents, as shown in Figure 6. The oxygen content of the deposits increases as the ratio of the residues of the original particles increases, and the small-sized aggregates decrease in the fractions collected (from high to low impactor stage numbers, in accordance with Figure 1). However, it must be noted that the oxygen detected with the SEM-EDS system may partly be bound to the carbon serving as the aerosol collector. Since the uncovered surface ratio of the impactor plates increases as larger and larger particles are collected (with decreasing amounts), the contribution of the oxygen present on the carbon surface may lead to an apparent increase in the O weight percentage. Even though this factor alone cannot be responsible for the trend shown in Figure 6 (the SEM-EDS analysis of the blank carbon surface shows much less oxygen; see Appendix E, Figure A6), a similar difference can be seen between the analysis results obtained for large and small surface areas. If small aggregates are analyzed locally with no uncovered C impactor surface, the result is usually much closer to 20 wt.%, i.e., to the value obtained for layers formed from aggregated particles. If a local analysis is carried out for spherical particles, the oxygen content is often relatively large, especially when the morphological features of the original powder can no longer be identified on the particles, indicating that they may have undergone in-depth oxidation during the laser treatment. In this respect, a clear difference can be seen between the residual particles that either do or do not exhibit the surface trenches resembling the original ones (those showing the surface trenches contain less oxygen than those whose surface is featureless).

### 3.3. Oxidation State of Metals Assessed via XANES

**Figure 7:**
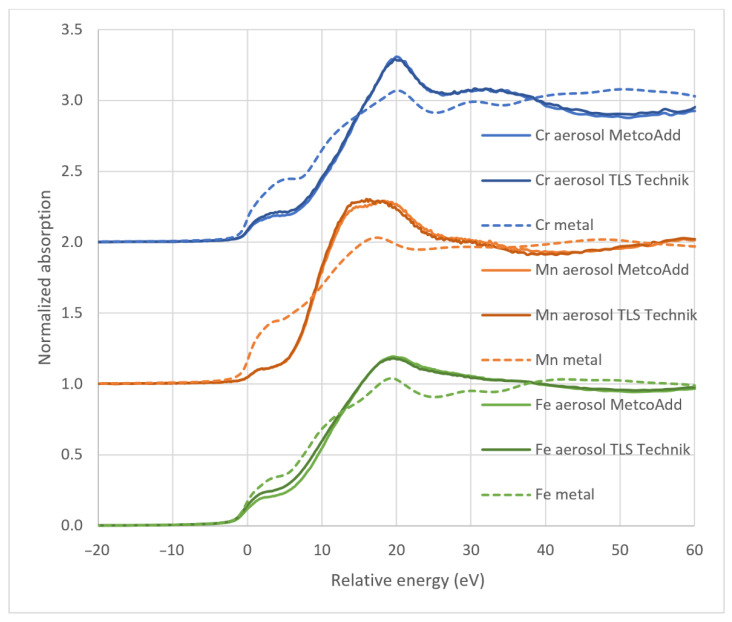
Comparison of Cr, Mn, and Fe normalized XANES spectra of total aerosols collected on a filter and those obtained for the corresponding reference metal foils.

The Cr, Mn, and Fe K-edge XANES spectra collected for the total aerosol samples and metals (stainless steel) are summarized in Figure 7. In order to compare the near-edge structures for different metals, the energy scale is displayed relative to the respective K-edges (i.e., 5989 eV for Cr, 6539 eV for Mn, and 7112 eV for Fe). The oxidation state can be derived from the chemical shift of the absorption edge towards positive energies for oxidized chemical forms. The peaks and oscillations near the absorption edges can also be used as fingerprints for specific chemical states, e.g., a specific sharp and narrow peak at 4.4 eV of relative energy is characteristic for Cr^6+^ compounds [44].

In general, the XANES spectra of the total aerosol generated from both types of H13 feedstock powders look similar for all selected metals. Since the edge features (at around 0 eV relative energy) are smaller with respect to the metallic spectra for Cr, Mn, and Fe, the presence of oxidized metals is expected to be significant in the aerosol samples (see Figure 7). For Mn, only a small step is visible at the absorption edge energy of the Mn metal (0 eV relative), indicating that Mn is highly oxidized. For Cr, the presence of Cr^6+^ could be excluded due to the absence of the characteristic pre-edge peak.

Quantitative information on the oxidation states of Cr, Mn, and Fe was obtained via LCF performed using the standard spectra for Cr, Fe, and Mn metal foils and reference compounds of higher oxidation states such as Cr^3+^and Cr^6+^, Mn^2+^ and Mn^3+^, as well as Fe^2+^ and Fe^3+^. The fitting results for the total aerosols are summarized in Table 3, while size-fractionated details are also shown for the TLS Technik H13 feedstock powder as a representative starting material.

All three studied metals were found to be oxidized significantly. The order of metals based on the oxidation degree was Mn > Cr > Fe (see Me^0^ values in Table 3); Mn was found to be totally oxidized. The oxidation number generally decreases with increasing particle sizes, almost reaching the metallic state for the largest particles studied. The particles collected at stage 3 are mostly fragments or satellite particles of the original feedstock powder grains but can have chains of ultrafine particles condensed on their surfaces. Combining these findings with the SMPS results, as the total aerosol is dominated by ultrafine particles representing more than 70% of the total mass, it becomes obvious that the ultrafine aerosol particles contain the highest fraction of oxidized metals.

### 3.4. SEM Analysis of the Particulate Residues of the Starting Powders

**Figure 8:**
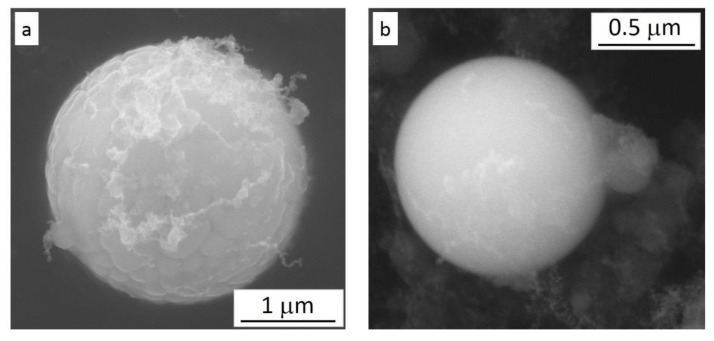
Representative images of the spherical particle types found on the impactor plates: (**a**) particle resembling the starting ones with respect to the surface morphology (also with filamentary deposits originating from the evaporated and oxidized metal vapor); (**b**) particle with an apparently even surface without any specific morphological characteristics.

Several stand-alone spherical particles measuring 250–2000 nm diameter outside the central deposit line of the impactor plates were observed for both starting materials and for all laser powers tested. The diameter of these particles is only slightly correlated with the nominal size range of the impactor. These particles were found to be ideally suited for individually analysis via SEM-EDS, since the excitation volume of the electron beam did not extend to the neighboring particles. The SEM images of two typical particles are shown in Figure 8. For particles whose diameter fell into the micrometer range, the surface features found for the original particles can still be seen (Figure 8a). This is very likely because either these particles are present already in the as-received metal powder or they are formed as the outer shell of the original evaporated particles, whilst the core remains intact. The surfaces of the smaller particles (Figure 8b) are rather uniform without any morphological characteristics. These particles usually contained more oxygen than those belonging to the previous category and exhibited a stronger deviation from the H13 standard composition.

**Figure 9:**
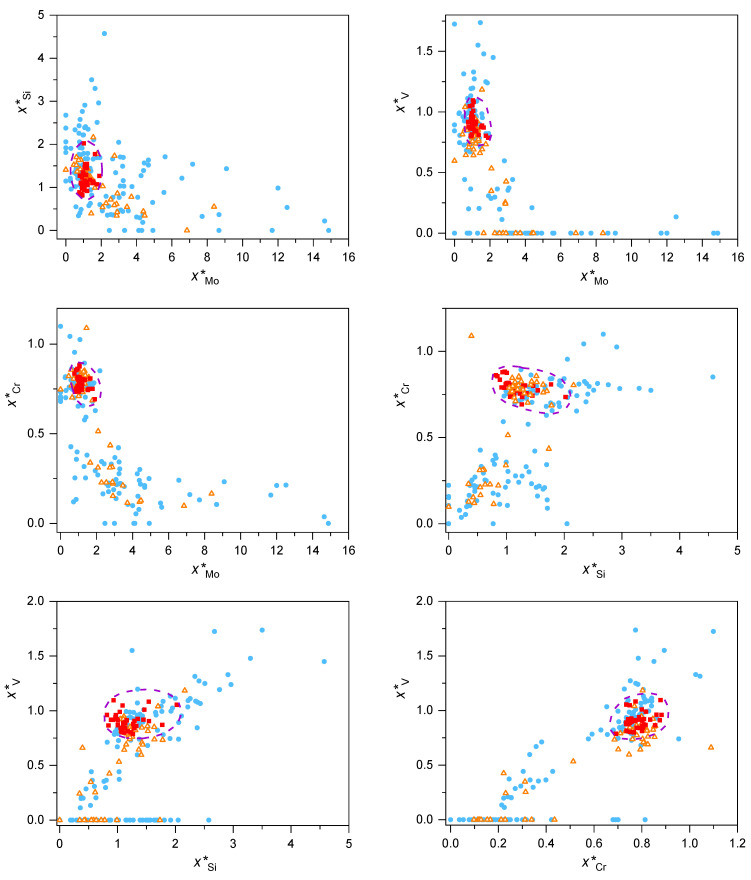
x*_Mc1_ vs. x*_Mc2_ functions obtained for three samples: blue circles—MetcoAdd powder processed at laser powers of 60, 85, and 100%; empty orange triangles—TLS Technik samples processed at 85% laser power; red squares—MetcoAdd powders either ground in a mortar or processed with the cladding instrument without operating the laser (i.e., with the particle stream only at ambient temperature). The areas marked with a dashed line represent the particles that are likely to pass the laser system without any composition change (no substantial composition deviation as compared to the unprocessed reference).

The composition analysis of the particles revealed that the Mn contents of the particles mostly could not be detected. Hence, Mn is no longer considered in the following analysis. The changes in concentration of the rest of the minor components (Mc) are highly correlated. To visualize these changes, x*_Mc1_ vs. x*_Mc2_ figures were constructed. The correlations obtained were nearly identical regardless of the materials, the laser power used the impactor stage where a specific particle was found, and even the particle size; therefore, all data can be displayed together. The data can be seen in Figure 9.

**Figure 10:**
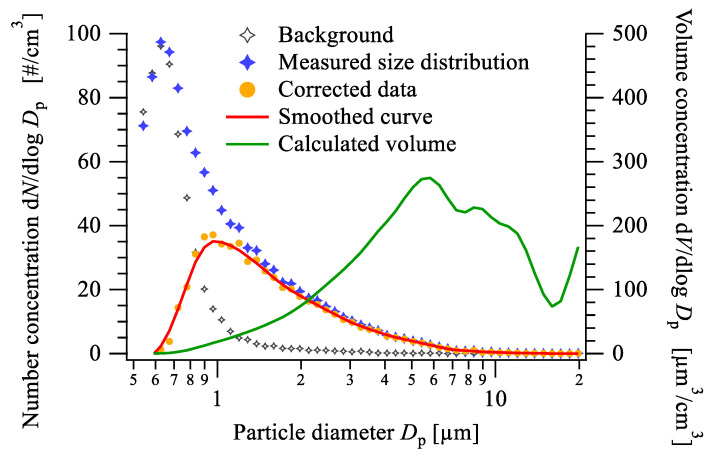
Measured size distribution of the released particles by the APS during laser cladding with the TLS H13 powder. The measured data (blue stars) were corrected with background concentrations (empty grey stars) in all size bins (orange dots). The green line shows the calculated volume size distribution, assuming that the particles are spherical. The APS measures the aerodynamic size, while the SEM images show the geometrical size of the particles.

On the basis of the mutual composition correlations, the following trends can be outlined. A large number of particles trapped on the impactor plates are identical to those found in the original powders. The composition of these particles can be seen in the marked areas in the graphs in Figure 9. It cannot be assessed whether these relatively small particles are present already in the as-received metal powder or form upon the evaporation of the outer shell of the larger precursor particles. The surface pattern identical to the original powders gives a hint that these particles are also present in the original powder, since the surface of the “satellite” particles of the original powders is rather smooth. This assumption is supported by the APS measurements. The size distribution measured by the APS shows a pronounced peak around 1 µm with an elongated tail towards larger particles (APS measurement shown in Figure 10). The time period elapsed between the measurements of the background and the new particles’ size distributions was 7 min long, which included the start of the cladding process followed by the stabilization of the environment. Aggregation processes are not fast enough to grow the ≈10–20 nm primary particles into this size range in the given time frame [45,46,47]. Furthermore, measurements were taken under identical conditions to the case when the system was producing a welded workpiece, but the laser was switched off. It was found that neither the APS nor the SMPS measured additional concentrations compared to the background, which means that the powder feeder did not provide extra particles in the size range covered by the two instruments (10 nm to 20 µm). As can be observed in the SEM images (Appendix B, Figure A2 and Figure A3), a large number of micron-sized particles are bound to the 45–90 µm starting grains. They are probably detached from the carrier grains via interactions with the laser. It has to be noted here that the real aerodynamic size of the satellite particles is 10 to 15% lower than the detected ones due to the effect of the particle density on the sizing of the APS [48].

Particles whose composition is outside of the areas labelled in Figure 9 are of modified composition. The difference in the composition distributions of the two starting materials is small; the only dissimilarity is that particles with high Mo and Si contents were found in the MetcoAdd samples only. It is possible that this starting material is less homogeneous at the micrometer scale, and local accumulations of Mo and Si are already present in the starting powder. In both samples, the Si content tends to decrease and the Mo content of the particles is relatively large (Figure 9, top left graph).

In order to determine the migration correspondence of each pair of studied elements, Spearman correlation coefficients were calculated, which is an excellent measure of a nonlinear monotonic relationship. Particles showing no sign of transformation and identified as original powder (i.e., falling in the labelled areas in Figure 9) were excluded, as well as particles lacking one of the two elements studied here. The Cr and V contents of the particle shows an interesting correlation with the Mo and Si contents. The high Cr and V losses can be associated with the large Mo content of the particles (R_(Cr,Mo)_ = –0.67 and R_(V,Mo)_ = –0.51), while the relatively high Si content is desired for V and Cr retention (R_(V,Si)_ = 0.91 and R_(Cr,Si)_ = 0.66). Although the concentration of all these elements is less than 5 wt.% in the Fe-rich matrix, it is interesting to note that both Cr and V are silicide-forming metals with several stable silicide phases, but they exhibit a nearly complete immiscibility with Mo. Since V and Cr are relatively volatile as compared to Si and Mo, it is not surprising that they selectively evaporated from the particles annealed by the cladding environment. The bottom right graph of Figure 9 indicates that these two elements migrate in a correlated manner (R_(V,Cr)_ = 0.88), and their loss occurs simultaneously.

Although the mean particle composition in the present study was measured with the SEM-EDS method only, the particles themselves might not be homogeneous. An in-depth composition analysis performed on individual particles showed [49] that (i) a significant component redistribution within the particles is indeed possible during the laser melting and (ii) surface oxidation may take place. In spite of the differences in either the powder composition or the analysis methods, the results of the present and the above-cited study are in line with each other.

## 4. Summary and Conclusions

We characterized two H13 steel powders and their aerosol products formed in the laser cladding process. To the best knowledge of the authors, this is the first comprehensive and comparative particle-level study of the laser cladding process with H13 alloys. The new findings can be summarized as follows:

- The chemical composition of the powder particles conformed to the H13 specification and the manufacturers’ datasheets. The morphological analysis showed that the powder particles were mostly spherical, but also elongated and irregularly shaped ones were present. The powder particles from the two vendors carried different irregularity types. Smaller micrometer-sized satellite particles with the same chemical composition were also observed, particularly in one of the powders;
- We characterized the particles formed in the laser cladding process via SEM-EDS. In line with previous results [19], it was found that the composition of the aerosol product changed compared to the as-received metal powder. Metals with higher vapor pressure were enriched and became oxidized significantly in the finest fraction of the sampled aerosol;
- The residues of the original particles, which were not incorporated into the welded part but were transferred with the aerosol, exhibited a composition change. The elements with relatively high vapor pressure (typically, V and Cr) escaped from the particle residues, while other elements with relatively low vapor pressure (e.g., Mo) accumulated. The concentration changes of the components were also correlated in the sense that a relatively high local Si content led to good retention of V and Cr, but a high local Mo content was linked to intense losses of V and Cr. The components missing from the residues of the metallic particles were found in the highly oxidized ultrafine aerosol;
- The mass size distributions of the aerosol samples collected with the cascade impactor showed good agreement with the results of the electromobility and aerodynamic size measurements, which were conducted using a scanning mobility particle spectrometer and an aerodynamic particle sizer. Our results show that the majority of the newly formed aerosol particles fall into the ultrafine region. The number size distribution follows the lognormal distribution with a peak value at slightly below 70 nm, while the maximum of the volume size distribution is at 200 nm;
- The morphology and chemical composition of the collected size-fractionated aerosol samples were determined via scanning electron microscopy, energy-dispersive spectroscopy, and a total-reflection X-ray fluorescence analysis. The size distributions of the elemental concentrations could be calculated from air concentrations for each size fraction determined via TXRF. The calculated size distribution of the iron, which is the balance in the composition of the H13 types steels, showed significant conformity with the double peak volume size distributions calculated from the number size distributions measured by the SMPS and the APS;
- The bimodality of the mass size distribution was observed in the size distributions of the minor constituents, but to different extents, in line with the enrichment of the particular element. Strong evidence was found that the two modes consist of the newly formed aerosol particles at around 200 nm and the predominantly metallic satellite particles at around 5 µm, both in the respirable size range. Based on the literature data, it can also be assumed that both modes contribute to the adverse effects on the fabrication process, especially health-related concerns.

As nano- and micrometer-sized metallic particles are potentially toxic or carcinogenic to humans, appropriate extraction devices and personal protection equipment are required when operating such machines. In addition, these metal particles can cause considerable extinction of the laser beam, affecting the cladding process itself.

## Figures and Tables

**Table 1:** Nominal composition of H13 and equivalent-grade tool steels according to US, European, and Japanese standards. Values are given in wt.%.

Grade: Standard:	H13 ASTM A681 [30]	1.2344/X40CrMoV5-1 EN ISO 4957: 2000 [31]	SKD61 JIS G4404 [32]
C	0.32–0.45	0.35–0.42	0.35–0.42
Si	0.8–1.25	0.8–1.2	0.8–1.2
P	<0.03	<0.03	<0.03
S	<0.03	<0.02	<0.02
V	0.8–1.2	0.85–1.15	0.8–1.15
Cr	4.75–5.5	4.8–5.5	4.8–5.5
Mn	0.2–0.6	0.25–0.5	0.25–0.5
Mo	1.1–1.75	1.0–1.5	1.1–1.5
Fe	Balance	Balance	Balance

**Table 2:** Cut-off size of the impactor plates at the 16.7 L/min flow rate used during our experiments.

Impactor Plate Number	Cut-Off Size of the Particles (µm)
1	17.9
2	8.9
3	4.5
4	2.25
5	1.13
6	0.57
7	0.29
8	0.18
9	0.07

**Table 3:** LCF results for Cr, Mn, and Fe K-edge XANES of total aerosol particles (mostly ultrafine particles) and 180–300 nm (stage 8) and 2.25–4.5 µm (stage 3) fractions collected during laser cladding *.

Sample → ↓ Element	MetcoAdd, Total Aerosol	TLS Technik, Total Aerosol	TLS Technik, Stage 8	TLS Technik, Stage 3
Cr^0^ fraction	0.29	0.36	0.40	0.82
Cr^3+^ fraction	0.71	0.64	0.60	0.18
Cr^6+^ fraction	–	–	–	–
**Cr mean oxidation number**	**+2.1**	**+1.9**	**+1.8**	**+0.5**
Mn^0^ fraction	–	–	0.01	–
Mn^2+^ fraction	0.57	0.63	0.85	–
Mn^3+^ fraction	0.43	0.37	0.14	–
**Mn mean oxidation number**	**+2.4**	**+2.4**	**+2.1**	–
Fe^0^ fraction	0.56	0.77	0.74	0.92
Fe^2+^ fraction	–	0.10	0.10	0.07
Fe^3+^ fraction	0.44	0.13	0.16	0.01
**Fe mean oxidation number**	**+1.3**	**+1.1**	**+0.7**	**+0.2**

* The uncertainty levels for oxidation state fractions and formal oxidation numbers are ±0.03 and ±0.1, respectively.

## Data Availability

The data related to the present study can be obtained upon personal request.

## Appendix A. Compositions of the Raw Materials and the Welded Objects

A comparison of the composition of as-received metal powders as measured via SEM-EDS is shown in Figure A1. Oxygen and carbon lines were excluded from the analysis because they may originate from the adsorbed impurities.

## Appendix B. Morphological Description of the Raw Materials

Figure A2 shows representative scanning electron micrographs of the two starting materials attached to adhesive carbon tapes. In both particle populations, nearly spherical and elongated ellipsoidal particles are present. The mean particle size is conformal to the nominal value provided by the vendors of the H13 powders. The surfaces of the particles are ribbed irregularly in both cases (see Figure A3).

The typical irregularities of the particles are different for the two starting materials. For MetcoAdd, the common defect type is the attachment of small particles (“bulges”) of identical surface morphology to the large particles. Attached round-shaped particles with a smooth surface and small diameter as compared to the major spheres are very scarce. However, among the particles in the TLS Technik powder, many small smooth spheres attached to the large particles can be identified. Additionally, some particles are covered with shells of dissimilar morphology as compared to the main particles (bottom left image in Figure A3). The attached smooth particles, as well as the shells, appear as originating from partial re-melting of a part of the as-received metal powder. The local composition at both above-mentioned irregularity types was identical for the major particles. Although it was assumed that most of the particles are dense, a few hollow particles could also be found in the TLS Technik sample (Figure A3, bottom right image).

In both particle populations studied, there were truncated particles that looked as if the originally spherical particles were damaged. The local morphologies at the truncated spots showed characteristic differences for the two sorts of starting materials (see the comparison in Figure A4). The surfaces of the concave parts of the MetcoAdd particles conformed to the rest of the particle surfaces, showing the same trench pattern. However, on the truncated parts of the TLS Technik powder, the trench-like morphological features disappeared, and these parts were densely populated with smooth and mostly perfectly spherical minor particles. Both above-mentioned features of the damaged parts of this powder type give a hint that the truncation is related to local melting.

## Appendix C. Morphology of the Fine-Grained Deposit on the Impactor Plates

The deposit collected on the impactor plates was obtained as an agglomeration of particles. These deposit-forming agglomerated particles were much smaller for each impactor stage than the cut-off size of the corresponding impactor stage. This fact indicates that the particle agglomeration started in the gas phase prior to the adhesion of the flying aerosol particles to the impactor plates.

Where the deposit makes a thick layer compared to the diameter of the units identified as agglomerated particles, the image usually cannot be resolved so that the primary particles can be identified. However, there are two cases where the primary particles can be easily visualized: (i) the mean deposit thickness is comparable to the primary particle size (see Figure A5, top images); (ii) the filament composed of the primary particles stands far off from the deposit and can be imaged by the SEM at a much different focus distance than the background (see Figure A5, bottom images).

## Appendix D. Vapor Pressure and Oxidation Heat of the Elemental Components of the H13 Alloy

The vapor pressure of the major metallic components and their oxidation heat are shown in Table A1. It can be seen that vanadium is the most volatile component among the major constituents, which explains why it melts the alloy very easily.

Additionally, as shown in Figure 9 of the main part of the paper, the vanadium concentration reaches the detection limit first, as shown in the graph of x*_V_ vs. x*_Cr_. This means that V escapes earlier from the particulate residues of the original powder than the next more volatile element, Cr.

As the oxidation heat data shows, vanadium is highly prone to oxidation. Since the vanadium concentration is below the detection limit for the deposit collected at impactor stage #9, it is very likely that the vanadium oxide is produced either very far from the collection zone or the particles are too small to be trapped on the impactor plates.

**Table A1:** Vapor pressure and oxidation heat values of the major metallic constituents of the H13 alloy. Data refer to 1200 °C in general, and oxidation heat values refer to one mole of the metal. The accuracy of the data is indicated by the number of significant digits shown.

Metal	Vapor Pressure /mbar	Oxidation State	Oxidation Heat /kJ moL^−1^
V	3.2 × 10^−2^	+3	−420
Mn	1.0 × 10^–3^	+3	−275
Cr	4.0 × 10^–7^	+3	−334
Fe	2.0 × 10^–7^	+2	−150

## Appendix E. SEM-EDS Analysis of the Carbon Tape Used as the Impactor Plate

The carbon tape used as the impactor plate was measured via SEM-EDS in order to assess the oxygen content of the background. The spectrum obtained is shown in Figure A6. The oxygen content of the carbon substrate was 20 wt.% (15.8 at.%).
